# Crystal structures of the sulfones of 2,3-diphenyl-2,3-di­hydro-4*H*-1,3-benzo­thia­zin-4-one and 2,3-diphenyl-2,3-di­hydro-4*H*-pyrido[3,2-*e*][1,3]thia­zin-4-one

**DOI:** 10.1107/S2056989023001524

**Published:** 2023-02-23

**Authors:** Hemant P. Yennawar, Tapas K. Mal, Carlos N. Pacheco, Anthony F. Lagalante, Mark A. Olsen, Michael W. Russell, Grace C. Muench, Quentin J. Moyer, Lee J. Silverberg

**Affiliations:** a Department of Biochemistry and Molecular Biology Pennsylvania State University, University Park, PA 16802, USA; b Department of Chemistry, The Pennsylvania State University, University Park, PA 16802, USA; c Mendel Science Center, Villanova University, 800 Lancaster Avenue, Villanova, PA 19085, USA; d Pennsylvania State University, Schuylkill Campus, 200 University Drive, Schuylkill Haven, PA 17972, USA; University of Aberdeen, United Kingdom

**Keywords:** sulfone, 1,3-thia­zin-4-one, screw-boat pucker, *S*-oxidation, crystal structure

## Abstract

The title sulfones, 2,3-diphenyl-2,3-di­hydro-4*H*-1,3-benzo­thia­zine-1,1,4-trione, C_20_H_15_NO_3_S, and 2,3-diphenyl-2,3-di­hydro-4*H*-pyrido[3,2-*e*][1,3]thia­zine-1,1,4-trione, C_19_H_14_N_2_O_3_S, crystallize in space group *P*2_1_/*n* with two mol­ecules in each of the asymmetric units and have almost identical unit cells and extended structures. In both structures, the thia­zine rings exhibit a screw-boat pucker. The inter­molecular inter­actions observed are C—H⋯O-type hydrogen bonds and parallel partial π–π stacking between the fused aromatic rings (benzo- or pyrido-) of the core of the mol­ecules within each asymmetric unit, and also connecting to mol­ecules with translational periodicity in the *a*-axis direction in what can be described as columns (two per asymmetric unit) of stacked mol­ecules with alternating chirality. The pendant phenyl groups of both mol­ecules do not participate in aromatic ring inter­actions.

## Abstract 2

The title sulfones crystallize in space group *P*2_1_/*n* with two mol­ecules in each of the asymmetric units and have almost identical unit cells and extended structures. In both structures, the thia­zine rings exhibit a screw-boat pucker.

## 1. Chemical context

The 2,3-di­hydro-4*H*-1,3-thia­zin-4-ones are a group of six-membered heterocycles with a wide range of biological activity (Ryabukhin *et al.*, 1996 ▸; Silverberg & Moyer, 2019 ▸). Surrey’s research (Surrey *et al.*, 1958 ▸, Surrey, 1963*a* ▸,*b* ▸) resulted in the discovery of two drugs, the anti­anxiety and muscle relaxant chlormezanone, 2-(4-chloro­phen­yl)-3-methyl-2,3,5,6-tetra­hydro-4*H*-1,3-thia­zin-4-one 1,1-dioxide (O’Neil, 2006 ▸; Tanaka & Hirayama, 2005 ▸) and the muscle relaxant dichlormezanone, 2-(3,4-di­chloro­phen­yl)-3-methyl-2,3,5,6-tetra­hydro-4*H*-1,3-thia­zin-4-one 1,1-dioxide (Elks & Ganellin, 1990 ▸). These sulfones showed greater activity than the sulfides from which they were synthesized (Surrey *et al.*, 1958 ▸). 
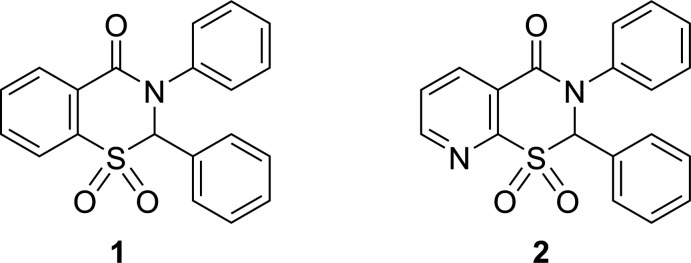


Compounds in this group with a fused benzene or pyridine ring are of particular inter­est because of their potential biological activity (Silverberg *et al.*, 2016 ▸, 2020 ▸, 2021 ▸; Malfara *et al.*, 2021 ▸). We have previously reported the preparation of the title sulfones 2,3-diphenyl-2,3-di­hydro-4*H*-1,3-benzo­thia­zin-4-one 1,1-dioxide **1** and 2,3-diphenyl-2,3-di­hydro-4*H*-pyrido[3,2-*e*][1,3]thia­zin-4-one 1,1-dioxide **2** (Silverberg, 2020 ▸). We have also described the X-ray crystal structures of the corresponding sulfides **3** and **4** and sulfoxides **5** and **6** (Yennawar, Bendinsky *et al.*, 2014 ▸; Yennawar, Singh *et al.*, 2014 ▸; Yennawar, Fox *et al.*, 2017 ▸; Yennawar, Noble *et al.*, 2017 ▸). Herein we report the crystal structures of **1** and **2**, along with a more complete characterization than previously reported (Silverberg, 2020 ▸).

## 2. Structural commentary

**Figure 1:**
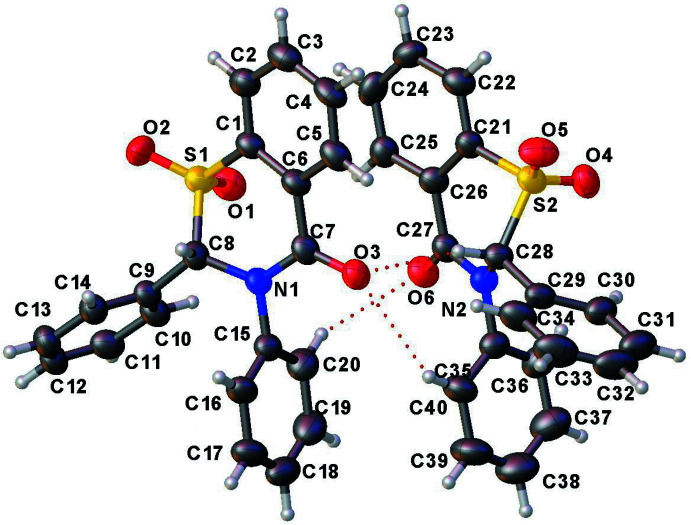
The asymmetric unit of **1** with displacement ellipsoids drawn at 50% probability level.

**Figure 3:**
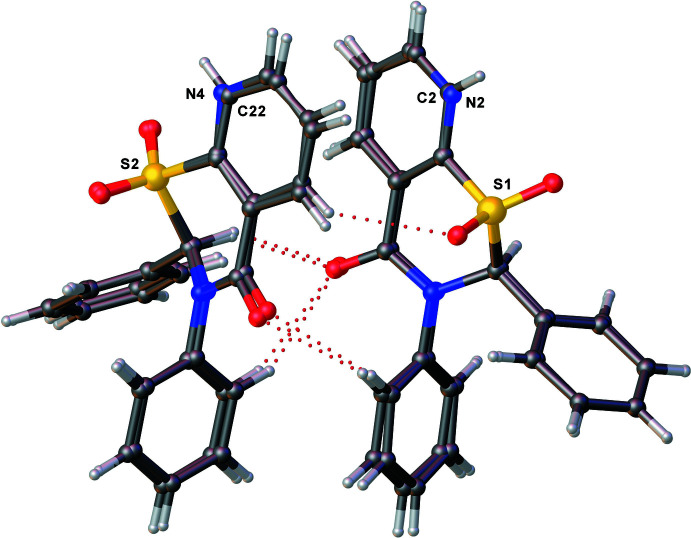
Overlay plot of **1** and **2** where atoms S1 and S2 of the two structures are matched. The atoms that differ in the two structures, namely C2 and C22 of **1** and N2 and N4 of **2**, are labeled.

The two title compounds (Figs. 1 ▸ and 2 ▸) are structurally very similar with two mol­ecules of each in the asymmetric units of the respective crystals. Both of the crystal structures are in the monoclinic space group *P*2_1_/*n* with very similar unit-cell dimensions, and are fairly well superimposable (Fig. 3 ▸).

The structures of **1** and **2** both display a screw-boat (pucker) conformation for the four thia­zine rings in the two asymmetric units [puckering amplitude *Q* ranging between 0.616 (4) and 0.6449 (16) Å, and the θ and φ values, after accounting for the absolute conformation transformations, are between 60.7 (4) and 63.02 (16)°, and 140.53 (18) and 142.9 (4)°, respectively]. The puckering observed is similar to that in the sulfoxides **5** and **6** (Yennawar, Fox *et al.* 2017 ▸; Yennawar, Noble *et al.* 2017 ▸), but is different from the envelope conformations seen in the sulfides **3** and **4** (Yennawar, Bendinsky *et al.*, 2014 ▸; Yennawar, Singh *et al.*, 2014 ▸). Each mol­ecule contains one stereogenic center, which lies between the N atom and the SO_2_ group of the heterocyclic ring: in the asymmetric unit of **1**, C8 has an *S* configuration and C28 an *R* configuration, thus generating a racemic pair. The situation is **2** is similar, with C8 *S* and C28 *R.*

In compound **1**, the dihedral angle between the substituent phenyl rings is 58.7 (2) and 57.4 (3)° in the two asymmetric mol­ecules. Between the co-planar atoms of the fused benzene and the phenyl rings, the dihedral angle ranges between 83 and 100°. Compound **2** is again similar, showing a dihedral angle between the substituent phenyl rings of 53.04 (11) and 57.24 (13)°. Between the co-planar atoms of the pyridine ring and the phenyl rings of the respective structures, the dihedral angle ranges between 76 and 101°.

## 3. Supra­molecular features

**Figure 4:**
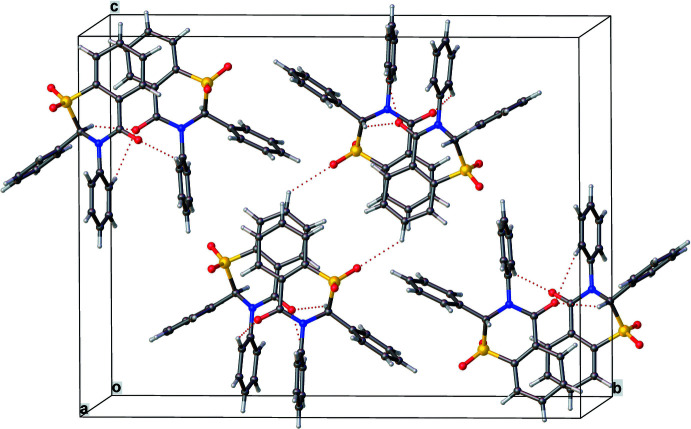
Crystal packing diagram for **1** showing inter­molecular pairs of C—H⋯O hydrogen bonds. Columns (four per unit cell) of mol­ecules with alternating chirality, due to the translational periodicity down the ***a*** direction can be envisioned.

Both of the extended structures are consolidated by C—H⋯O hydrogen bonds (Tables 1 ▸ and 2 ▸). Assuming that the C—H⋯O angle should be greater than or equal to 130° as one of the qualifiers for such inter­actions, a very small variation in mol­ecular positioning in the two structures results in two additional inter­actions for **2** as compared to that in **1**. However, in both structures (starting from the inter­actions within the asymmetric units followed by translation periodicity along the ***a*** direction) inter­molecular π–π stacking inter­actions between the fused aromatic rings, namely the benzene ring of the benzo­thia­zine unit in **1** and the pyridine ring of the pyrido­thia­zine unit in **2**, can be seen (Figs. 4 ▸ and 5 ▸). The centroid–centroid separations in **1** are 3.522 (3) and 3.521 (3) Å with corresponding values of 3.5715 (15) and 3.5991 (15) Å in **2**.

## 4. Database survey

Searches were undertaken using the American Chemical Society’s Chemical Abstract Service (CAS) Scifinder platform. Only two crystal structures of other sulfones of 2,3-di­hydro-4*H*-1,3-benzo­thia­zin-4-ones have been reported (Elghamry *et al.* 2007 ▸). No other sulfone of a 2,3-di­hydro-4*H*-pyrido[3,2-*e*][1,3]thia­zin-4-one has been synthesized.

## 5. Synthesis and crystallization

TLC plates (silica gel GF, 250-micron, 10 × 20 cm, cat. No. 21521) were purchased from Analtech. TLCs were visualized under short-wave UV, and then with I_2_, and then by spraying with ceric ammonium nitrate/sulfuric acid and heating. Infrared spectra were run on a Thermo-Fisher NICOLET iS50 FT–IR using a diamond-ATR attachment for direct powder analysis (Penn State Schuylkill). ^1^H and ^13^C NMR experiments (Penn State’s shared NMR facility, University Park) were carried out on a Bruker Advance-III-HD 500.20-MHz (^1^H frequency) instrument using a 5 mm Prodigy (liquid nitro­gen cooled) BBO BB-1H/19F/D Z-GRD cryoprobe. Samples were dissolved in CDCl_3_ and analyzed at room temperature. Typical conditions for ^1^H acquisition were 2 s relaxation delay, acquisition time of 4.089 s, spectral width of 8 kHz, 16 scans. Spectra were zero-filled to 128k points, and multiplied by exponential multiplication (EM with LB = 0.3 Hz) prior to FT. For the ^13^C experiments, data were acquired with power-gated ^1^H decoupling using a 2 s relaxation delay, with acquisition time of 1.1 s, spectral width of 29.8 kHz, and 256 scans. Spectra were zero-filled once, and multiplied by EM with LB = 2 Hz prior to FT. The exact masses of the synthesized compounds were determined using LC–MS (Villanova University). Exact mass was measured on a SCIEX Exion LC with a SCIEX 5600+ TripleTOF MS. Separation was achieved on an Agilent Infinity LabPoroshell 120 EC-C18 column maintained at 40°C with a gradient of 90/10 (water/aceto­nitrile with 0.1% formic acid) ramped from 5/95 over 6 min at a flowrate of 0.5 ml min^−1^. The TOF–MS was scanned over 100–500 Da and calibrated with the SCIEX APCI positive calibrant solution prior to accurate mass analysis. Compound exact mass was measured in positive ESI mode with a DP = 100 V, CE = 10, GAS1 = GAS2 = 60 psi, CUR = 30 psi, ISV = 5500 V, and source temperature of 450°C. Melting points were performed on a Vernier Melt Station (Penn State Schuylkill).

**General Oxidation Procedure** (Surrey *et al.*, 1958 ▸; Silverberg, 2020 ▸; Cannon *et al.*, 2015 ▸): The heterocycle (0.267 mmol) was dissolved in glacial acetic acid (1.2 ml). An aqueous solution of KMnO_4_ (0.535 mmol in 1.45 ml water) was added dropwise at room temperature with vigorous stirring. The reaction was followed by TLC. Solid sodium bis­ulfite (NaHSO_3_/Na_2_S_2_O_5_) was added until the solution remained colorless; 1.45 ml of water was added and stirred for 10 min. The mixture was extracted with CH_2_Cl_2_ (3 × 5 ml). The organics were combined and washed once with sat. NaCl. The solution was dried over Na_2_SO_4_ and filtered. The product was purified by chromatography in a silica gel micro-column with mixtures of ethyl acetate and hexa­nes. Crystals for X-ray were grown as detailed below.

2,3-Diphenyl-2,3-di­hydro-4*H*-1,3-benzo­thia­zin-4-one 1,1-dioxide, **1**: Crystals for X-ray analysis were grown by slow evaporation from 2-propanol solution. 0.050 g (54%). m.p. 436–438 K. ^1^H NMR (CDCl_3_): δ(ppm): 8.29 (*d*, 1H, *J* = 7.7 Hz), 7.70 (*m*, 2H), 7.60 (*t*, *J* = 7.6 Hz, 1H), 7.32 (*m*, 2H), 7.25 (*m*, 8H), 5.79 (*m*, 1H, C2-H). ^13^C NMR (CDCl_3_) δ(ppm) 161.1 (C=O), 141.9, 135.0, 134.1, 133.4, 130.6, 130.0, 129.8, 129.1, 128.9, 128.6, 128.2, 126.6, 124.1, 82.6 (C2). HRMS *(m*/*z*): [*M* + H]+ of 350.0838 is consistent with calculated [*M* + H]+ of 350.0845. IR (neat, cm^−1^): 1655 (C=O), 1313 (SO_2_). *R*_f_ (50% EtOAc/hexa­nes) = 0.46.

2,3-Diphenyl-2,3-di­hydro-4*H*-pyrido[3,2-*e*][1,3]thia­zin-4-one 1,1-dioxide, **2**: Crystals for X-ray were grown by slow evaporation from ethanol solution. 0.079 g (77%). m.p.: 483–484 K (decomposition). ^1^H NMR (CDCl_3_): δ(ppm): 8.77 (*d*, *J* = 5.1 Hz, 1H), 8.62 (*d*, *J* = 8.0 Hz, 1H), 7.67 (*dd*, *J* = 8.1, 4.7 Hz, 1H), 7.31 (*m*, 9H), 5.88 (*s*, 1H, C2-H). ^13^C NMR (CDCl_3_) δ(ppm): 160.5 (C=O), 153.9, 152.2, 141.3, 139.0, 130.2, 129.8, 129.1, 128.7, 128.4, 128.3, 126.4, 82.4 (C2). HRMS *(m*/*z*): [*M* + H]+ of 351.0790 is consistent with calculated [*M* + H]+ of 351.0797. IR (neat, cm^−1^): 1655 (C=O), 1325 (SO_2_). *R*_f_ (50% EtOAc/hexa­nes) = 0.37.

## 6. Refinement

Crystal data, data collection and structure refinement details are summarized in Table 3 ▸. The hydrogen atoms were placed in their geometrically calculated positions and refined using the riding model with parent-atom—H lengths of 0.93–0.95 Å (aromatic CH) and 0.98–1.00 Å (methine CH). Isotropic displacement parameters for these atoms were set to 1.2 times *U*_eq_ of the parent atom.

## Supplementary Material

Crystal structure: contains datablock(s) general, 2, 1. DOI: 10.1107/S2056989023001524/hb8054sup1.cif

Structure factors: contains datablock(s) 1. DOI: 10.1107/S2056989023001524/hb80541sup2.hkl

Supporting information file. DOI: 10.1107/S2056989023001524/hb80541sup4.mol

Structure factors: contains datablock(s) 2. DOI: 10.1107/S2056989023001524/hb80542sup3.hkl

Supporting information file. DOI: 10.1107/S2056989023001524/hb80542sup5.mol

Supporting information file. DOI: 10.1107/S2056989023001524/hb80541sup6.cml

Supporting information file. DOI: 10.1107/S2056989023001524/hb80542sup7.cml

CCDC references: 2236814, 2236815

Additional supporting information:  crystallographic information; 3D view; checkCIF report

## Figures and Tables

**Figure 2:**
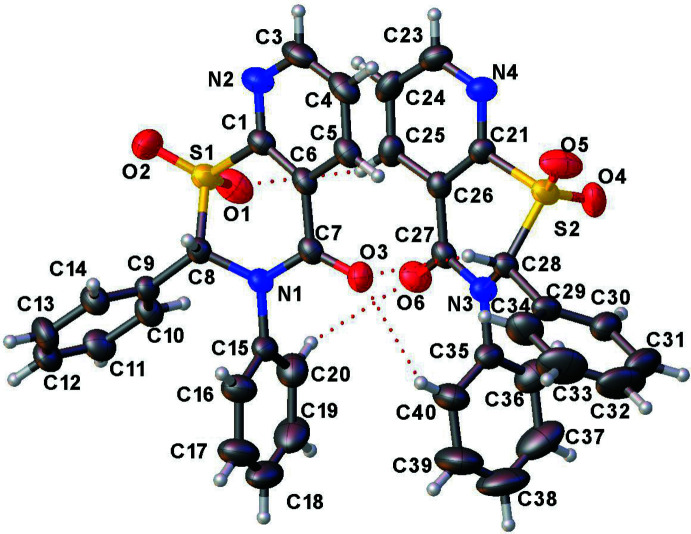
The asymmetric unit of **2** with displacement ellipsoids drawn at 50% probability level.

**Figure 5:**
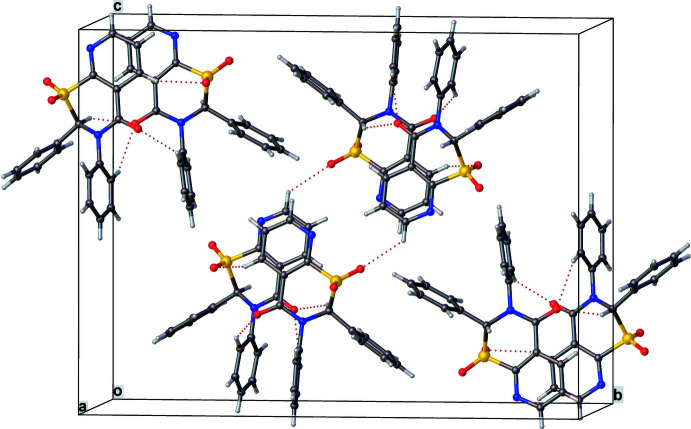
Crystal packing diagram for **2** showing strong similarity with that of **1**.

**Table 1:** Hydrogen-bond geometry (Å, °) for **1** [Chem scheme1]

*D*—H⋯*A*	*D*—H	H⋯*A*	*D*⋯*A*	*D*—H⋯*A*
C3—H3⋯O5^i^	0.95	2.65	3.353 (6)	131
C8—H8⋯O6^ii^	1.00	2.29	3.070 (6)	134
C16—H16⋯O6^ii^	0.95	2.67	3.410 (6)	135
C20—H20⋯O6	0.95	2.61	3.422 (6)	144
C28—H28⋯O3	1.00	2.31	3.096 (5)	135
C36—H36⋯O3^iii^	0.95	2.62	3.412 (6)	141
C40—H40⋯O3	0.95	2.73	3.473 (7)	136

Symmetry codes: (i) 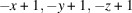 ; (ii) 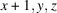 ; (iii) 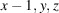 .

**Table 2:** Hydrogen-bond geometry (Å, °) for **2** [Chem scheme1]

*D*—H⋯*A*	*D*—H	H⋯*A*	*D*⋯*A*	*D*—H⋯*A*
C3—H3⋯O5^i^	0.93	2.56	3.234 (3)	130
C5—H5⋯O4^ii^	0.93	2.69	3.388 (2)	132
C8—H8⋯O6^ii^	0.98	2.31	3.089 (2)	136
C16—H16⋯O6^ii^	0.93	2.71	3.466 (3)	139
C20—H20⋯O6	0.93	2.70	3.496 (3)	145
C25—H25⋯O1	0.93	2.75	3.463 (3)	135
C28—H28⋯O3	0.98	2.37	3.126 (2)	134
C36—H36⋯O3^iii^	0.93	2.64	3.381 (3)	137
C40—H40⋯O3	0.93	2.68	3.439 (3)	140

Symmetry codes: (i) 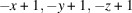 ; (ii) 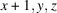 ; (iii) 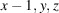 .

**Table 3:** Experimental details

	**1**	**2**
Crystal data		
Chemical formula	C_20_H_15_NO_3_S	C_19_H_14_N_2_O_3_S
*M*_r_	349.39	350.38
Crystal system, space group	Monoclinic, *P*2_1_/*n*	Monoclinic, *P*2_1_/*n*
Temperature (K)	173	298
*a*, *b*, *c* (Å)	6.8530 (6), 25.7472 (15), 19.0240 (12)	6.8584 (19), 25.487 (7), 19.008 (5)
β (°)	97.394 (7)	94.669 (7)
*V* (Å^3^)	3328.8 (4)	3311.6 (16)
*Z*	8	8
Radiation type	Cu *K*α	Mo *K*α
μ (mm^−1^)	1.89	0.22
Crystal size (mm)	0.2 × 0.06 × 0.04	0.22 × 0.04 × 0.02

Data collection		
Diffractometer	ROD, Synergy Custom system, HyPix-Arc 150	Bruker CCD area detector
Absorption correction	Multi-scan (*CrysAlis PRO*; Rigaku OD, 2022 ▸)	Multi-scan (*SADABS*; Krause *et al.*, 2015 ▸)
*T*_min_, *T*_max_	0.649, 1.000	0.237, 0.9
No. of measured, independent and observed [*I* > 2σ(*I*)] reflections	21581, 6557, 4087	29524, 7909, 5509
*R*_int_	0.074	0.040
(sin θ/λ)_max_ (Å^−1^)	0.628	0.668

Refinement		
*R*[*F* ^2^ > 2σ(*F* ^2^)], *wR*(*F* ^2^), *S*	0.092, 0.300, 1.10	0.051, 0.138, 1.04
No. of reflections	6557	7909
No. of parameters	452	451
H-atom treatment	H-atom parameters constrained	H-atom parameters constrained
Δρ_max_, Δρ_min_ (e Å^−3^)	0.66, −1.06	0.31, −0.27

Computer programs: *CrysAlis PRO* (Rigaku OD, 2022 ▸), *SMART* (Bruker, 2001 ▸), *SAINT* (Bruker, 2016 ▸), *OLEX2.solve* (Bourhis *et al.*, 2015 ▸), *SHELXS* (Sheldrick, 2008 ▸), *SHELXL2018/3* (Sheldrick, 2015 ▸), *OLEX2* and (Dolomanov *et al.*, 2009 ▸).

## supplementary crystallographic information

### 2,3-Diphenyl-2,3-dihydro-4H-1,3-benzothiazine-1,1,4-trione (1) Crystal data

**Table:**

C_20_H_15_NO_3_S	*F*(000) = 1456
*M**_r_* = 349.39	*D*_x_ = 1.394 Mg m^−^^3^
Monoclinic, *P*2_1_/*n*	Cu *K*α radiation, λ = 1.54184 Å
*a* = 6.8530 (6) Å	Cell parameters from 6963 reflections
*b* = 25.7472 (15) Å	θ = 2.9–70.6°
*c* = 19.0240 (12) Å	µ = 1.89 mm^−^^1^
β = 97.394 (7)°	*T* = 173 K
*V* = 3328.8 (4) Å^3^	Block, clear colourless
*Z* = 8	0.2 × 0.06 × 0.04 mm

### 2,3-Diphenyl-2,3-dihydro-4H-1,3-benzothiazine-1,1,4-trione (1) Data collection

**Table:**

ROD, Synergy Custom system, HyPix-Arc 150 diffractometer	6557 independent reflections
Radiation source: Rotating-anode X-ray tube, Rigaku (Cu) X-ray Source	4087 reflections with *I* > 2σ(*I*)
Mirror monochromator	*R*_int_ = 0.074
Detector resolution: 10.0000 pixels mm^-1^	θ_max_ = 75.7°, θ_min_ = 2.9°
ω scans	*h* = −8→8
Absorption correction: multi-scan (CrysAlisPro; Rigaku OD, 2022)	*k* = −31→31
*T*_min_ = 0.649, *T*_max_ = 1.000	*l* = −23→18
21581 measured reflections	

### 2,3-Diphenyl-2,3-dihydro-4H-1,3-benzothiazine-1,1,4-trione (1) Refinement

**Table:**

Refinement on *F*^2^	Hydrogen site location: inferred from neighbouring sites
Least-squares matrix: full	H-atom parameters constrained
*R*[*F*^2^ > 2σ(*F*^2^)] = 0.092	*w* = 1/[σ^2^(*F*_o_^2^) + (0.1722*P*)^2^ + 1.2363*P*] where *P* = (*F*_o_^2^ + 2*F*_c_^2^)/3
*wR*(*F*^2^) = 0.300	(Δ/σ)_max_ < 0.001
*S* = 1.10	Δρ_max_ = 0.66 e Å^−^^3^
6557 reflections	Δρ_min_ = −1.06 e Å^−^^3^
452 parameters	Extinction correction: SHELXL2018/3 (Sheldrick 2015), Fc^*^=kFc[1+0.001xFc^2^λ^3^/sin(2θ)]^-1/4^
0 restraints	Extinction coefficient: 0.0038 (5)
Primary atom site location: iterative	

### 2,3-Diphenyl-2,3-dihydro-4H-1,3-benzothiazine-1,1,4-trione (1) Special details

**Table:**

Geometry. All esds (except the esd in the dihedral angle between two l.s. planes) are estimated using the full covariance matrix. The cell esds are taken into account individually in the estimation of esds in distances, angles and torsion angles; correlations between esds in cell parameters are only used when they are defined by crystal symmetry. An approximate (isotropic) treatment of cell esds is used for estimating esds involving l.s. planes.

### 2,3-Diphenyl-2,3-dihydro-4H-1,3-benzothiazine-1,1,4-trione (1) Fractional atomic coordinates and isotropic or equivalent isotropic displacement parameters (Å^2^)

**Table:**

	*x*	*y*	*z*	*U*_iso_*/*U*_eq_	
S1	0.62556 (19)	0.74906 (4)	0.62956 (6)	0.0454 (4)	
O1	0.4248 (5)	0.76270 (12)	0.63479 (18)	0.0532 (9)	
O2	0.7399 (6)	0.78342 (13)	0.59272 (17)	0.0608 (10)	
O3	0.5497 (5)	0.61116 (11)	0.74902 (16)	0.0456 (8)	
N1	0.6484 (5)	0.69574 (13)	0.74977 (18)	0.0390 (8)	
C1	0.6386 (7)	0.68627 (17)	0.5944 (2)	0.0407 (10)	
C2	0.6537 (7)	0.6796 (2)	0.5231 (3)	0.0501 (12)	
H2	0.654648	0.708677	0.492500	0.060*	
C3	0.6673 (8)	0.6295 (2)	0.4972 (3)	0.0544 (13)	
H3	0.680789	0.624314	0.448640	0.065*	
C4	0.6615 (7)	0.58719 (19)	0.5413 (3)	0.0517 (12)	
H4	0.668259	0.553102	0.522656	0.062*	
C5	0.6457 (7)	0.59407 (18)	0.6128 (3)	0.0489 (12)	
H5	0.643583	0.564708	0.642941	0.059*	
C6	0.6330 (7)	0.64378 (17)	0.6404 (2)	0.0419 (10)	
C7	0.6090 (7)	0.64872 (16)	0.7171 (2)	0.0424 (10)	
C8	0.7557 (7)	0.73578 (16)	0.7164 (2)	0.0428 (10)	
H8	0.887282	0.721153	0.709704	0.051*	
C9	0.7905 (7)	0.78609 (15)	0.7569 (2)	0.0399 (10)	
C10	0.6351 (7)	0.81491 (17)	0.7762 (2)	0.0464 (11)	
H10	0.504192	0.802207	0.766774	0.056*	
C11	0.6728 (9)	0.86284 (18)	0.8097 (2)	0.0537 (13)	
H11	0.566833	0.883072	0.822377	0.064*	
C12	0.8631 (8)	0.88091 (18)	0.8244 (3)	0.0515 (12)	
H12	0.888316	0.913247	0.847853	0.062*	
C13	1.0162 (8)	0.85202 (19)	0.8050 (3)	0.0533 (13)	
H13	1.147062	0.864694	0.814852	0.064*	
C14	0.9815 (8)	0.80463 (18)	0.7713 (2)	0.0490 (12)	
H14	1.088108	0.784884	0.758093	0.059*	
C15	0.6318 (7)	0.69896 (16)	0.8253 (2)	0.0429 (11)	
C16	0.7980 (8)	0.69680 (18)	0.8737 (2)	0.0516 (12)	
H16	0.923333	0.691947	0.858343	0.062*	
C17	0.7812 (10)	0.7018 (2)	0.9453 (3)	0.0638 (16)	
H17	0.895753	0.700715	0.979190	0.077*	
C18	0.6001 (11)	0.7083 (2)	0.9673 (3)	0.0691 (18)	
H18	0.589298	0.712069	1.016297	0.083*	
C19	0.4346 (10)	0.7092 (2)	0.9185 (3)	0.0669 (16)	
H19	0.309207	0.713126	0.934068	0.080*	
C20	0.4482 (8)	0.70451 (19)	0.8461 (3)	0.0537 (12)	
H20	0.333554	0.705118	0.812218	0.064*	
S2	0.13252 (18)	0.49892 (4)	0.65164 (6)	0.0439 (4)	
O4	−0.0701 (5)	0.48580 (12)	0.65590 (18)	0.0540 (9)	
O5	0.2516 (6)	0.46129 (12)	0.62200 (17)	0.0565 (10)	
O6	0.0546 (5)	0.64719 (11)	0.74688 (16)	0.0467 (8)	
N2	0.1453 (6)	0.56260 (13)	0.76148 (18)	0.0408 (9)	
C21	0.1453 (7)	0.55831 (17)	0.6066 (2)	0.0413 (10)	
C22	0.1579 (7)	0.55769 (18)	0.5345 (2)	0.0481 (11)	
H22	0.161209	0.525749	0.509711	0.058*	
C23	0.1655 (8)	0.6047 (2)	0.4994 (2)	0.0506 (12)	
H23	0.174775	0.605013	0.449995	0.061*	
C24	0.1596 (7)	0.65089 (18)	0.5357 (3)	0.0506 (12)	
H24	0.164357	0.682885	0.511127	0.061*	
C25	0.1468 (7)	0.65103 (18)	0.6081 (2)	0.0461 (11)	
H25	0.145517	0.683064	0.632868	0.055*	
C26	0.1357 (7)	0.60427 (17)	0.6446 (2)	0.0403 (10)	
C27	0.1111 (7)	0.60690 (16)	0.7219 (2)	0.0420 (10)	
C28	0.2553 (7)	0.51938 (16)	0.7368 (2)	0.0419 (10)	
H28	0.387925	0.532827	0.729278	0.050*	
C29	0.2872 (8)	0.47302 (16)	0.7859 (2)	0.0454 (11)	
C30	0.1322 (9)	0.44441 (18)	0.8061 (2)	0.0556 (14)	
H30	0.000299	0.454607	0.790836	0.067*	
C31	0.1698 (11)	0.4010 (2)	0.8486 (3)	0.0698 (17)	
H31	0.063439	0.381662	0.862724	0.084*	
C32	0.3619 (12)	0.3856 (2)	0.8706 (3)	0.078 (2)	
H32	0.386987	0.355338	0.898849	0.093*	
C33	0.5153 (11)	0.4141 (2)	0.8514 (3)	0.0766 (19)	
H33	0.646738	0.404008	0.867541	0.092*	
C34	0.4806 (9)	0.4575 (2)	0.8087 (3)	0.0590 (14)	
H34	0.587839	0.476665	0.794842	0.071*	
C35	0.1235 (7)	0.56603 (16)	0.8364 (2)	0.0434 (11)	
C36	−0.0597 (8)	0.5610 (2)	0.8570 (3)	0.0567 (13)	
H36	−0.172083	0.556851	0.822632	0.068*	
C37	−0.0791 (10)	0.5620 (2)	0.9288 (3)	0.0699 (16)	
H37	−0.205174	0.558456	0.944078	0.084*	
C38	0.0863 (11)	0.5680 (3)	0.9778 (3)	0.0791 (19)	
H38	0.073055	0.568055	1.026936	0.095*	
C39	0.2698 (11)	0.5741 (3)	0.9570 (3)	0.0789 (19)	
H39	0.381975	0.578891	0.991238	0.095*	
C40	0.2890 (9)	0.5731 (2)	0.8853 (2)	0.0570 (13)	
H40	0.414579	0.577332	0.869928	0.068*	

### 2,3-Diphenyl-2,3-dihydro-4H-1,3-benzothiazine-1,1,4-trione (1) Atomic displacement parameters (Å^2^)

**Table:**

	*U*^11^	*U*^22^	*U*^33^	*U*^12^	*U*^13^	*U*^23^
S1	0.0655 (9)	0.0296 (6)	0.0391 (6)	−0.0001 (5)	−0.0002 (5)	0.0001 (4)
O1	0.061 (2)	0.0395 (17)	0.057 (2)	0.0105 (16)	−0.0017 (16)	−0.0030 (15)
O2	0.097 (3)	0.0413 (19)	0.0443 (18)	−0.0202 (18)	0.0095 (18)	0.0079 (15)
O3	0.058 (2)	0.0301 (15)	0.0467 (17)	−0.0032 (14)	0.0004 (14)	−0.0002 (13)
N1	0.053 (2)	0.0259 (17)	0.0377 (19)	−0.0017 (15)	0.0038 (16)	−0.0035 (14)
C1	0.050 (3)	0.033 (2)	0.039 (2)	0.0015 (19)	0.0021 (18)	−0.0064 (18)
C2	0.057 (3)	0.048 (3)	0.042 (3)	−0.005 (2)	−0.003 (2)	0.000 (2)
C3	0.059 (3)	0.057 (3)	0.046 (3)	0.000 (3)	0.001 (2)	−0.013 (2)
C4	0.060 (3)	0.044 (3)	0.050 (3)	0.000 (2)	0.002 (2)	−0.021 (2)
C5	0.056 (3)	0.035 (2)	0.053 (3)	0.003 (2)	−0.004 (2)	−0.008 (2)
C6	0.055 (3)	0.032 (2)	0.038 (2)	0.0004 (19)	0.0021 (19)	−0.0066 (18)
C7	0.054 (3)	0.024 (2)	0.048 (3)	0.0027 (19)	0.002 (2)	−0.0033 (18)
C8	0.060 (3)	0.025 (2)	0.043 (2)	−0.0033 (19)	0.007 (2)	−0.0053 (18)
C9	0.056 (3)	0.0244 (19)	0.039 (2)	0.0002 (18)	0.0016 (19)	−0.0020 (17)
C10	0.059 (3)	0.032 (2)	0.046 (3)	0.001 (2)	−0.003 (2)	−0.003 (2)
C11	0.080 (4)	0.035 (2)	0.044 (3)	0.005 (2)	0.000 (2)	−0.001 (2)
C12	0.075 (4)	0.030 (2)	0.048 (3)	−0.010 (2)	0.001 (2)	−0.005 (2)
C13	0.061 (3)	0.042 (3)	0.055 (3)	−0.013 (2)	0.001 (2)	−0.004 (2)
C14	0.061 (3)	0.038 (2)	0.047 (3)	−0.007 (2)	0.002 (2)	−0.001 (2)
C15	0.062 (3)	0.027 (2)	0.039 (2)	−0.003 (2)	0.006 (2)	−0.0033 (18)
C16	0.069 (3)	0.043 (3)	0.039 (2)	−0.005 (2)	−0.007 (2)	0.005 (2)
C17	0.102 (5)	0.047 (3)	0.038 (3)	−0.004 (3)	−0.006 (3)	0.001 (2)
C18	0.128 (6)	0.038 (3)	0.043 (3)	−0.012 (3)	0.020 (3)	−0.003 (2)
C19	0.097 (5)	0.047 (3)	0.063 (3)	−0.009 (3)	0.033 (3)	−0.014 (3)
C20	0.067 (3)	0.042 (3)	0.055 (3)	0.000 (2)	0.017 (2)	−0.009 (2)
S2	0.0639 (8)	0.0257 (5)	0.0396 (6)	0.0007 (5)	−0.0026 (5)	−0.0015 (4)
O4	0.063 (2)	0.0390 (17)	0.057 (2)	−0.0106 (16)	−0.0041 (16)	0.0020 (15)
O5	0.087 (3)	0.0370 (17)	0.0442 (18)	0.0210 (17)	0.0040 (17)	−0.0065 (14)
O6	0.060 (2)	0.0281 (15)	0.0503 (18)	0.0065 (14)	0.0026 (15)	−0.0040 (14)
N2	0.056 (2)	0.0279 (17)	0.0372 (19)	−0.0005 (16)	0.0035 (16)	0.0005 (15)
C21	0.051 (3)	0.031 (2)	0.039 (2)	0.0002 (19)	−0.0025 (19)	0.0034 (18)
C22	0.059 (3)	0.041 (2)	0.043 (2)	0.006 (2)	0.001 (2)	−0.006 (2)
C23	0.061 (3)	0.052 (3)	0.038 (2)	0.005 (2)	0.004 (2)	0.008 (2)
C24	0.058 (3)	0.037 (2)	0.056 (3)	0.001 (2)	0.004 (2)	0.016 (2)
C25	0.053 (3)	0.034 (2)	0.050 (3)	0.000 (2)	0.000 (2)	0.004 (2)
C26	0.050 (3)	0.032 (2)	0.038 (2)	0.0057 (18)	−0.0014 (18)	0.0016 (18)
C27	0.052 (3)	0.029 (2)	0.043 (2)	−0.0015 (19)	−0.0007 (19)	0.0006 (19)
C28	0.060 (3)	0.030 (2)	0.035 (2)	0.001 (2)	0.0034 (19)	−0.0011 (18)
C29	0.071 (3)	0.028 (2)	0.035 (2)	0.003 (2)	−0.002 (2)	−0.0009 (18)
C30	0.091 (4)	0.035 (2)	0.041 (3)	−0.004 (2)	0.007 (2)	0.002 (2)
C31	0.125 (6)	0.038 (3)	0.046 (3)	−0.002 (3)	0.012 (3)	0.003 (2)
C32	0.142 (7)	0.042 (3)	0.045 (3)	0.018 (4)	−0.004 (4)	0.009 (2)
C33	0.109 (5)	0.057 (3)	0.057 (3)	0.023 (4)	−0.018 (3)	0.004 (3)
C34	0.079 (4)	0.043 (3)	0.049 (3)	0.011 (3)	−0.013 (3)	0.000 (2)
C35	0.062 (3)	0.030 (2)	0.039 (2)	0.003 (2)	0.008 (2)	0.0006 (18)
C36	0.064 (3)	0.053 (3)	0.054 (3)	0.003 (3)	0.011 (2)	0.009 (2)
C37	0.087 (5)	0.069 (4)	0.057 (3)	0.013 (3)	0.022 (3)	0.008 (3)
C38	0.114 (6)	0.076 (4)	0.050 (3)	0.006 (4)	0.021 (4)	−0.005 (3)
C39	0.106 (5)	0.088 (5)	0.040 (3)	−0.006 (4)	−0.003 (3)	−0.007 (3)
C40	0.074 (4)	0.055 (3)	0.039 (3)	−0.005 (3)	−0.004 (2)	−0.005 (2)

### 2,3-Diphenyl-2,3-dihydro-4H-1,3-benzothiazine-1,1,4-trione (1) Geometric parameters (Å, º)

**Table:**

S1—O1	1.436 (4)	S2—O4	1.442 (4)
S1—O2	1.425 (3)	S2—O5	1.429 (3)
S1—C1	1.756 (4)	S2—C21	1.760 (4)
S1—C8	1.806 (5)	S2—C28	1.805 (4)
O3—C7	1.239 (5)	O6—C27	1.224 (5)
N1—C7	1.372 (5)	N2—C27	1.370 (5)
N1—C8	1.459 (5)	N2—C28	1.455 (5)
N1—C15	1.458 (5)	N2—C35	1.454 (5)
C1—C2	1.383 (6)	C21—C22	1.385 (6)
C1—C6	1.405 (6)	C21—C26	1.393 (6)
C2—H2	0.9500	C22—H22	0.9500
C2—C3	1.387 (7)	C22—C23	1.386 (6)
C3—H3	0.9500	C23—H23	0.9500
C3—C4	1.378 (7)	C23—C24	1.380 (7)
C4—H4	0.9500	C24—H24	0.9500
C4—C5	1.390 (6)	C24—C25	1.392 (6)
C5—H5	0.9500	C25—H25	0.9500
C5—C6	1.390 (6)	C25—C26	1.397 (6)
C6—C7	1.494 (6)	C26—C27	1.502 (6)
C8—H8	1.0000	C28—H28	1.0000
C8—C9	1.510 (5)	C28—C29	1.514 (6)
C9—C10	1.386 (6)	C29—C30	1.387 (7)
C9—C14	1.387 (7)	C29—C34	1.399 (7)
C10—H10	0.9500	C30—H30	0.9500
C10—C11	1.398 (6)	C30—C31	1.383 (7)
C11—H11	0.9500	C31—H31	0.9500
C11—C12	1.379 (7)	C31—C32	1.387 (9)
C12—H12	0.9500	C32—H32	0.9500
C12—C13	1.375 (7)	C32—C33	1.370 (9)
C13—H13	0.9500	C33—H33	0.9500
C13—C14	1.385 (6)	C33—C34	1.383 (7)
C14—H14	0.9500	C34—H34	0.9500
C15—C16	1.370 (6)	C35—C36	1.369 (7)
C15—C20	1.375 (7)	C35—C40	1.383 (6)
C16—H16	0.9500	C36—H36	0.9500
C16—C17	1.388 (7)	C36—C37	1.391 (7)
C17—H17	0.9500	C37—H37	0.9500
C17—C18	1.370 (9)	C37—C38	1.381 (9)
C18—H18	0.9500	C38—H38	0.9500
C18—C19	1.370 (9)	C38—C39	1.375 (9)
C19—H19	0.9500	C39—H39	0.9500
C19—C20	1.397 (7)	C39—C40	1.387 (7)
C20—H20	0.9500	C40—H40	0.9500
			
O1—S1—C1	110.4 (2)	O4—S2—C21	109.8 (2)
O1—S1—C8	110.4 (2)	O4—S2—C28	111.0 (2)
O2—S1—O1	118.4 (2)	O5—S2—O4	117.8 (2)
O2—S1—C1	109.0 (2)	O5—S2—C21	109.3 (2)
O2—S1—C8	109.2 (2)	O5—S2—C28	109.2 (2)
C1—S1—C8	97.5 (2)	C21—S2—C28	97.9 (2)
C7—N1—C8	120.4 (4)	C27—N2—C28	121.2 (4)
C7—N1—C15	117.5 (4)	C27—N2—C35	117.1 (4)
C15—N1—C8	119.8 (3)	C35—N2—C28	119.0 (3)
C2—C1—S1	120.2 (4)	C22—C21—S2	119.0 (3)
C2—C1—C6	121.6 (4)	C22—C21—C26	122.5 (4)
C6—C1—S1	118.2 (3)	C26—C21—S2	118.5 (3)
C1—C2—H2	120.6	C21—C22—H22	120.7
C1—C2—C3	118.7 (5)	C21—C22—C23	118.6 (4)
C3—C2—H2	120.6	C23—C22—H22	120.7
C2—C3—H3	119.7	C22—C23—H23	119.8
C4—C3—C2	120.6 (4)	C24—C23—C22	120.4 (4)
C4—C3—H3	119.7	C24—C23—H23	119.8
C3—C4—H4	119.8	C23—C24—H24	119.7
C3—C4—C5	120.5 (4)	C23—C24—C25	120.5 (4)
C5—C4—H4	119.8	C25—C24—H24	119.7
C4—C5—H5	119.9	C24—C25—H25	119.9
C4—C5—C6	120.2 (5)	C24—C25—C26	120.3 (4)
C6—C5—H5	119.9	C26—C25—H25	119.9
C1—C6—C7	123.9 (4)	C21—C26—C25	117.7 (4)
C5—C6—C1	118.3 (4)	C21—C26—C27	124.4 (4)
C5—C6—C7	117.8 (4)	C25—C26—C27	117.9 (4)
O3—C7—N1	121.6 (4)	O6—C27—N2	122.1 (4)
O3—C7—C6	119.8 (4)	O6—C27—C26	119.9 (4)
N1—C7—C6	118.6 (4)	N2—C27—C26	117.9 (4)
S1—C8—H8	107.6	S2—C28—H28	107.4
N1—C8—S1	108.3 (3)	N2—C28—S2	108.2 (3)
N1—C8—H8	107.6	N2—C28—H28	107.4
N1—C8—C9	116.0 (4)	N2—C28—C29	116.3 (4)
C9—C8—S1	109.3 (3)	C29—C28—S2	109.7 (3)
C9—C8—H8	107.6	C29—C28—H28	107.4
C10—C9—C8	121.2 (4)	C30—C29—C28	122.3 (5)
C10—C9—C14	120.1 (4)	C30—C29—C34	119.4 (5)
C14—C9—C8	118.6 (4)	C34—C29—C28	118.2 (5)
C9—C10—H10	120.3	C29—C30—H30	120.0
C9—C10—C11	119.4 (5)	C31—C30—C29	119.9 (6)
C11—C10—H10	120.3	C31—C30—H30	120.0
C10—C11—H11	119.8	C30—C31—H31	119.8
C12—C11—C10	120.3 (5)	C30—C31—C32	120.4 (6)
C12—C11—H11	119.8	C32—C31—H31	119.8
C11—C12—H12	120.1	C31—C32—H32	120.1
C13—C12—C11	119.8 (5)	C33—C32—C31	119.8 (5)
C13—C12—H12	120.1	C33—C32—H32	120.1
C12—C13—H13	119.7	C32—C33—H33	119.7
C12—C13—C14	120.6 (5)	C32—C33—C34	120.6 (6)
C14—C13—H13	119.7	C34—C33—H33	119.7
C9—C14—H14	120.1	C29—C34—H34	120.1
C13—C14—C9	119.7 (5)	C33—C34—C29	119.8 (6)
C13—C14—H14	120.1	C33—C34—H34	120.1
C16—C15—N1	119.7 (4)	C36—C35—N2	119.2 (4)
C16—C15—C20	121.6 (5)	C36—C35—C40	121.5 (5)
C20—C15—N1	118.7 (4)	C40—C35—N2	119.3 (4)
C15—C16—H16	120.4	C35—C36—H36	120.4
C15—C16—C17	119.3 (5)	C35—C36—C37	119.2 (5)
C17—C16—H16	120.4	C37—C36—H36	120.4
C16—C17—H17	119.9	C36—C37—H37	120.3
C18—C17—C16	120.3 (5)	C38—C37—C36	119.4 (6)
C18—C17—H17	119.9	C38—C37—H37	120.3
C17—C18—H18	120.0	C37—C38—H38	119.3
C17—C18—C19	119.9 (5)	C39—C38—C37	121.3 (6)
C19—C18—H18	120.0	C39—C38—H38	119.3
C18—C19—H19	119.6	C38—C39—H39	120.4
C18—C19—C20	120.8 (6)	C38—C39—C40	119.2 (6)
C20—C19—H19	119.6	C40—C39—H39	120.4
C15—C20—C19	118.2 (5)	C35—C40—C39	119.4 (6)
C15—C20—H20	120.9	C35—C40—H40	120.3
C19—C20—H20	120.9	C39—C40—H40	120.3
			
S1—C1—C2—C3	178.8 (4)	S2—C21—C22—C23	179.2 (4)
S1—C1—C6—C5	−179.3 (4)	S2—C21—C26—C25	179.8 (3)
S1—C1—C6—C7	2.4 (6)	S2—C21—C26—C27	−1.2 (7)
S1—C8—C9—C10	65.3 (5)	S2—C28—C29—C30	−62.1 (5)
S1—C8—C9—C14	−111.2 (4)	S2—C28—C29—C34	114.8 (4)
O1—S1—C1—C2	98.3 (4)	O4—S2—C21—C22	−95.0 (4)
O1—S1—C1—C6	−81.6 (4)	O4—S2—C21—C26	82.9 (4)
O1—S1—C8—N1	55.2 (3)	O4—S2—C28—N2	−56.2 (3)
O1—S1—C8—C9	−72.2 (4)	O4—S2—C28—C29	71.6 (4)
O2—S1—C1—C2	−33.3 (5)	O5—S2—C21—C22	35.7 (4)
O2—S1—C1—C6	146.8 (4)	O5—S2—C21—C26	−146.4 (4)
O2—S1—C8—N1	−173.1 (3)	O5—S2—C28—N2	172.3 (3)
O2—S1—C8—C9	59.6 (4)	O5—S2—C28—C29	−59.9 (4)
N1—C8—C9—C10	−57.5 (6)	N2—C28—C29—C30	61.0 (6)
N1—C8—C9—C14	125.9 (5)	N2—C28—C29—C34	−122.0 (5)
N1—C15—C16—C17	177.7 (4)	N2—C35—C36—C37	177.2 (5)
N1—C15—C20—C19	−178.0 (4)	N2—C35—C40—C39	−177.1 (5)
C1—S1—C8—N1	−59.9 (3)	C21—S2—C28—N2	58.7 (3)
C1—S1—C8—C9	172.8 (3)	C21—S2—C28—C29	−173.5 (4)
C1—C2—C3—C4	1.5 (8)	C21—C22—C23—C24	−0.3 (8)
C1—C6—C7—O3	159.3 (4)	C21—C26—C27—O6	−161.4 (4)
C1—C6—C7—N1	−18.8 (7)	C21—C26—C27—N2	15.7 (7)
C2—C1—C6—C5	0.8 (7)	C22—C21—C26—C25	−2.4 (7)
C2—C1—C6—C7	−177.5 (4)	C22—C21—C26—C27	176.6 (4)
C2—C3—C4—C5	−1.3 (8)	C22—C23—C24—C25	0.3 (8)
C3—C4—C5—C6	0.9 (8)	C23—C24—C25—C26	−1.3 (7)
C4—C5—C6—C1	−0.6 (7)	C24—C25—C26—C21	2.3 (7)
C4—C5—C6—C7	177.8 (4)	C24—C25—C26—C27	−176.8 (4)
C5—C6—C7—O3	−19.0 (7)	C25—C26—C27—O6	17.6 (7)
C5—C6—C7—N1	162.8 (4)	C25—C26—C27—N2	−165.3 (4)
C6—C1—C2—C3	−1.3 (7)	C26—C21—C22—C23	1.4 (8)
C7—N1—C8—S1	56.4 (5)	C27—N2—C28—S2	−57.3 (5)
C7—N1—C8—C9	179.8 (4)	C27—N2—C28—C29	178.8 (4)
C7—N1—C15—C16	100.3 (5)	C27—N2—C35—C36	83.8 (5)
C7—N1—C15—C20	−80.1 (5)	C27—N2—C35—C40	−97.7 (5)
C8—S1—C1—C2	−146.7 (4)	C28—S2—C21—C22	149.2 (4)
C8—S1—C1—C6	33.4 (4)	C28—S2—C21—C26	−32.9 (4)
C8—N1—C7—O3	167.2 (4)	C28—N2—C27—O6	−165.2 (4)
C8—N1—C7—C6	−14.7 (6)	C28—N2—C27—C26	17.7 (6)
C8—N1—C15—C16	−62.6 (5)	C28—N2—C35—C36	−114.4 (5)
C8—N1—C15—C20	117.0 (5)	C28—N2—C35—C40	64.1 (6)
C8—C9—C10—C11	−176.0 (4)	C28—C29—C30—C31	177.1 (4)
C8—C9—C14—C13	176.6 (4)	C28—C29—C34—C33	−177.5 (5)
C9—C10—C11—C12	−1.0 (7)	C29—C30—C31—C32	−0.6 (8)
C10—C9—C14—C13	0.0 (7)	C30—C29—C34—C33	−0.5 (7)
C10—C11—C12—C13	1.0 (7)	C30—C31—C32—C33	1.4 (9)
C11—C12—C13—C14	−0.5 (7)	C31—C32—C33—C34	−1.7 (9)
C12—C13—C14—C9	0.0 (7)	C32—C33—C34—C29	1.2 (8)
C14—C9—C10—C11	0.5 (7)	C34—C29—C30—C31	0.2 (7)
C15—N1—C7—O3	4.4 (7)	C35—N2—C27—O6	−3.8 (7)
C15—N1—C7—C6	−177.5 (4)	C35—N2—C27—C26	179.1 (4)
C15—N1—C8—S1	−141.2 (3)	C35—N2—C28—S2	141.6 (3)
C15—N1—C8—C9	−17.8 (6)	C35—N2—C28—C29	17.7 (6)
C15—C16—C17—C18	0.7 (8)	C35—C36—C37—C38	0.1 (8)
C16—C15—C20—C19	1.5 (7)	C36—C35—C40—C39	1.4 (8)
C16—C17—C18—C19	0.7 (8)	C36—C37—C38—C39	1.2 (10)
C17—C18—C19—C20	−1.0 (8)	C37—C38—C39—C40	−1.1 (10)
C18—C19—C20—C15	−0.1 (7)	C38—C39—C40—C35	−0.1 (9)
C20—C15—C16—C17	−1.8 (7)	C40—C35—C36—C37	−1.3 (8)

### 2,3-Diphenyl-2,3-dihydro-4H-1,3-benzothiazine-1,1,4-trione (1) Hydrogen-bond geometry (Å, º)

**Table:**

*D*—H···*A*	*D*—H	H···*A*	*D*···*A*	*D*—H···*A*
C3—H3···O5^i^	0.95	2.65	3.353 (6)	131
C8—H8···O6^ii^	1.00	2.29	3.070 (6)	134
C16—H16···O6^ii^	0.95	2.67	3.410 (6)	135
C20—H20···O6	0.95	2.61	3.422 (6)	144
C28—H28···O3	1.00	2.31	3.096 (5)	135
C36—H36···O3^iii^	0.95	2.62	3.412 (6)	141
C40—H40···O3	0.95	2.73	3.473 (7)	136

Symmetry codes: (i) −*x*+1, −*y*+1, −*z*+1; (ii) *x*+1, *y*, *z*; (iii) *x*−1, *y*, *z*.

### 2,3-Diphenyl-2,3-dihydro-4H-pyrido[3,2-e][1,3]thiazine-1,1,4-trione (2) Crystal data

**Table:**

C_19_H_14_N_2_O_3_S	*F*(000) = 1456
*M**_r_* = 350.38	*D*_x_ = 1.406 Mg m^−^^3^
Monoclinic, *P*2_1_/*n*	Mo *K*α radiation, λ = 0.71073 Å
*a* = 6.8584 (19) Å	Cell parameters from 5480 reflections
*b* = 25.487 (7) Å	θ = 2.3–26.1°
*c* = 19.008 (5) Å	µ = 0.22 mm^−^^1^
β = 94.669 (7)°	*T* = 298 K
*V* = 3311.6 (16) Å^3^	Needle, colorless
*Z* = 8	0.22 × 0.04 × 0.02 mm

### 2,3-Diphenyl-2,3-dihydro-4H-pyrido[3,2-e][1,3]thiazine-1,1,4-trione (2) Data collection

**Table:**

Bruker CCD area detector diffractometer	5509 reflections with *I* > 2σ(*I*)
phi and ω scans	*R*_int_ = 0.040
Absorption correction: multi-scan (SADABS; Krause *et al.*, 2015)	θ_max_ = 28.4°, θ_min_ = 1.3°
*T*_min_ = 0.237, *T*_max_ = 0.9	*h* = −9→9
29524 measured reflections	*k* = −32→32
7909 independent reflections	*l* = −23→24

### 2,3-Diphenyl-2,3-dihydro-4H-pyrido[3,2-e][1,3]thiazine-1,1,4-trione (2) Refinement

**Table:**

Refinement on *F*^2^	Primary atom site location: structure-invariant direct methods
Least-squares matrix: full	Hydrogen site location: inferred from neighbouring sites
*R*[*F*^2^ > 2σ(*F*^2^)] = 0.051	H-atom parameters constrained
*wR*(*F*^2^) = 0.138	*w* = 1/[σ^2^(*F*_o_^2^) + (0.0689*P*)^2^ + 0.239*P*] where *P* = (*F*_o_^2^ + 2*F*_c_^2^)/3
*S* = 1.03	(Δ/σ)_max_ = 0.001
7909 reflections	Δρ_max_ = 0.31 e Å^−^^3^
451 parameters	Δρ_min_ = −0.26 e Å^−^^3^
0 restraints	

### 2,3-Diphenyl-2,3-dihydro-4H-pyrido[3,2-e][1,3]thiazine-1,1,4-trione (2) Special details

**Table:**

Experimental. The data collection nominally covered a full sphere of reciprocal space by a combination of 4 sets of ω scans each set at different φ and/or 2θ angles and each scan (10 s exposure) covering -0.300° degrees in ω. The crystal to detector distance was 5.82 cm.
Geometry. All esds (except the esd in the dihedral angle between two l.s. planes) are estimated using the full covariance matrix. The cell esds are taken into account individually in the estimation of esds in distances, angles and torsion angles; correlations between esds in cell parameters are only used when they are defined by crystal symmetry. An approximate (isotropic) treatment of cell esds is used for estimating esds involving l.s. planes.

### 2,3-Diphenyl-2,3-dihydro-4H-pyrido[3,2-e][1,3]thiazine-1,1,4-trione (2) Fractional atomic coordinates and isotropic or equivalent isotropic displacement parameters (Å^2^)

**Table:**

	*x*	*y*	*z*	*U*_iso_*/*U*_eq_	
S1	0.63430 (8)	0.74816 (2)	0.62562 (3)	0.04230 (15)	
O1	0.4325 (2)	0.75929 (6)	0.63311 (8)	0.0578 (4)	
O2	0.7474 (3)	0.78399 (6)	0.58785 (8)	0.0681 (5)	
O3	0.5672 (2)	0.60846 (5)	0.74613 (7)	0.0460 (3)	
N1	0.6591 (2)	0.69392 (5)	0.74620 (8)	0.0339 (3)	
C1	0.6539 (3)	0.68391 (7)	0.59048 (10)	0.0372 (4)	
N2	0.6667 (3)	0.68078 (7)	0.52113 (9)	0.0485 (4)	
C3	0.6840 (3)	0.63236 (9)	0.49538 (11)	0.0536 (6)	
H3	0.693463	0.628667	0.447104	0.064*	
C4	0.6885 (3)	0.58773 (8)	0.53596 (11)	0.0484 (5)	
H4	0.702610	0.554955	0.515465	0.058*	
C5	0.6718 (3)	0.59221 (7)	0.60787 (11)	0.0409 (5)	
H5	0.672293	0.562470	0.636234	0.049*	
C6	0.6543 (3)	0.64206 (7)	0.63705 (10)	0.0342 (4)	
C7	0.6251 (3)	0.64642 (7)	0.71450 (10)	0.0348 (4)	
C8	0.7641 (3)	0.73481 (6)	0.71038 (9)	0.0329 (4)	
H8	0.892706	0.720657	0.701537	0.039*	
C9	0.7973 (3)	0.78510 (6)	0.75095 (9)	0.0349 (4)	
C10	0.6433 (3)	0.81482 (7)	0.77271 (11)	0.0434 (5)	
H10	0.515080	0.803440	0.763175	0.052*	
C11	0.6813 (4)	0.86115 (8)	0.80844 (11)	0.0521 (6)	
H11	0.578441	0.880859	0.823488	0.062*	
C12	0.8701 (4)	0.87842 (8)	0.82198 (12)	0.0566 (6)	
H12	0.894905	0.909751	0.846138	0.068*	
C13	1.0224 (4)	0.84948 (9)	0.79988 (12)	0.0586 (6)	
H13	1.150197	0.861340	0.808938	0.070*	
C14	0.9866 (3)	0.80271 (8)	0.76418 (11)	0.0465 (5)	
H14	1.090055	0.783227	0.749150	0.056*	
C15	0.6365 (3)	0.69882 (7)	0.82159 (10)	0.0386 (4)	
C16	0.7981 (4)	0.69689 (8)	0.86865 (11)	0.0536 (6)	
H16	0.920527	0.689583	0.853283	0.064*	
C17	0.7772 (5)	0.70596 (10)	0.93957 (13)	0.0721 (7)	
H17	0.886765	0.705311	0.971784	0.087*	
C18	0.5986 (5)	0.71578 (10)	0.96239 (14)	0.0763 (9)	
H18	0.586778	0.722023	1.010056	0.092*	
C19	0.4354 (5)	0.71657 (10)	0.91580 (16)	0.0763 (8)	
H19	0.313012	0.722843	0.931876	0.092*	
C20	0.4533 (3)	0.70791 (9)	0.84404 (13)	0.0576 (6)	
H20	0.343538	0.708267	0.811932	0.069*	
S2	0.13994 (8)	0.49436 (2)	0.65360 (2)	0.03987 (15)	
O4	−0.0638 (2)	0.48498 (5)	0.66084 (8)	0.0538 (4)	
O5	0.2549 (3)	0.45389 (5)	0.62563 (8)	0.0642 (5)	
O6	0.0749 (2)	0.64799 (5)	0.73881 (7)	0.0468 (4)	
N3	0.1540 (2)	0.56289 (5)	0.75909 (8)	0.0356 (4)	
C21	0.1620 (3)	0.55275 (6)	0.60387 (10)	0.0348 (4)	
N4	0.1748 (2)	0.54607 (6)	0.53518 (8)	0.0432 (4)	
C23	0.1843 (3)	0.58965 (8)	0.49718 (11)	0.0485 (5)	
H23	0.191493	0.586542	0.448712	0.058*	
C24	0.1841 (3)	0.63940 (8)	0.52628 (11)	0.0469 (5)	
H24	0.192899	0.668864	0.497855	0.056*	
C25	0.1706 (3)	0.64482 (7)	0.59793 (11)	0.0410 (5)	
H25	0.169557	0.677987	0.618283	0.049*	
C26	0.1587 (2)	0.60021 (6)	0.63938 (9)	0.0327 (4)	
C27	0.1291 (3)	0.60600 (7)	0.71683 (10)	0.0354 (4)	
C28	0.2623 (3)	0.51705 (6)	0.73556 (9)	0.0340 (4)	
H28	0.392932	0.529097	0.725573	0.041*	
C29	0.2885 (3)	0.47234 (7)	0.78757 (10)	0.0416 (5)	
C30	0.1352 (4)	0.44247 (8)	0.80797 (11)	0.0533 (6)	
H30	0.007471	0.450892	0.791761	0.064*	
C31	0.1710 (5)	0.40003 (9)	0.85245 (13)	0.0733 (8)	
H31	0.067265	0.379812	0.865727	0.088*	
C32	0.3580 (6)	0.38766 (11)	0.87699 (14)	0.0930 (11)	
H32	0.381106	0.359142	0.907028	0.112*	
C33	0.5112 (5)	0.41710 (12)	0.85749 (15)	0.0914 (10)	
H33	0.638235	0.408655	0.874614	0.110*	
C34	0.4785 (4)	0.45936 (9)	0.81249 (13)	0.0633 (6)	
H34	0.583346	0.479040	0.798928	0.076*	
C35	0.1232 (3)	0.56794 (7)	0.83321 (10)	0.0422 (5)	
C36	−0.0608 (4)	0.55993 (9)	0.85463 (13)	0.0618 (6)	
H36	−0.165756	0.553010	0.821831	0.074*	
C37	−0.0874 (5)	0.56238 (12)	0.92666 (17)	0.0913 (10)	
H37	−0.210699	0.556903	0.942374	0.110*	
C38	0.0697 (7)	0.57292 (14)	0.97419 (16)	0.1078 (12)	
H38	0.052867	0.573858	1.022227	0.129*	
C39	0.2486 (6)	0.58194 (15)	0.95149 (15)	0.1071 (12)	
H39	0.353065	0.589783	0.984069	0.129*	
C40	0.2778 (4)	0.57965 (10)	0.88050 (12)	0.0692 (7)	
H40	0.400987	0.585986	0.865134	0.083*	

### 2,3-Diphenyl-2,3-dihydro-4H-pyrido[3,2-e][1,3]thiazine-1,1,4-trione (2) Atomic displacement parameters (Å^2^)

**Table:**

	*U*^11^	*U*^22^	*U*^33^	*U*^12^	*U*^13^	*U*^23^
S1	0.0610 (4)	0.0304 (2)	0.0346 (3)	0.0002 (2)	−0.0017 (2)	−0.00136 (19)
O1	0.0600 (10)	0.0494 (8)	0.0608 (10)	0.0194 (7)	−0.0144 (8)	−0.0116 (7)
O2	0.1144 (14)	0.0454 (9)	0.0452 (9)	−0.0225 (9)	0.0100 (9)	0.0039 (7)
O3	0.0600 (9)	0.0318 (7)	0.0461 (8)	−0.0101 (6)	0.0053 (7)	−0.0014 (6)
N1	0.0438 (9)	0.0268 (7)	0.0309 (8)	−0.0005 (6)	0.0024 (7)	−0.0046 (6)
C1	0.0402 (11)	0.0361 (9)	0.0349 (10)	0.0008 (8)	0.0009 (8)	−0.0062 (8)
N2	0.0591 (11)	0.0516 (10)	0.0347 (9)	−0.0033 (8)	0.0032 (8)	−0.0088 (8)
C3	0.0576 (14)	0.0645 (14)	0.0385 (12)	−0.0032 (11)	0.0019 (10)	−0.0208 (11)
C4	0.0447 (12)	0.0473 (11)	0.0531 (13)	−0.0001 (9)	0.0036 (10)	−0.0232 (10)
C5	0.0395 (11)	0.0353 (10)	0.0473 (12)	0.0008 (8)	−0.0003 (9)	−0.0110 (8)
C6	0.0326 (10)	0.0308 (9)	0.0389 (10)	−0.0009 (7)	0.0012 (8)	−0.0095 (7)
C7	0.0355 (10)	0.0298 (9)	0.0385 (10)	0.0002 (7)	−0.0008 (8)	−0.0051 (8)
C8	0.0362 (10)	0.0277 (8)	0.0344 (10)	0.0000 (7)	0.0011 (8)	−0.0026 (7)
C9	0.0458 (11)	0.0249 (8)	0.0334 (10)	−0.0018 (7)	0.0002 (8)	−0.0012 (7)
C10	0.0490 (12)	0.0327 (9)	0.0477 (12)	0.0019 (8)	−0.0012 (9)	−0.0064 (8)
C11	0.0706 (16)	0.0340 (10)	0.0513 (13)	0.0069 (10)	0.0034 (11)	−0.0091 (9)
C12	0.0846 (18)	0.0377 (11)	0.0478 (13)	−0.0140 (11)	0.0078 (12)	−0.0137 (10)
C13	0.0616 (15)	0.0550 (13)	0.0587 (15)	−0.0228 (11)	0.0017 (12)	−0.0157 (11)
C14	0.0502 (13)	0.0427 (11)	0.0466 (12)	−0.0043 (9)	0.0038 (10)	−0.0091 (9)
C15	0.0544 (12)	0.0278 (9)	0.0342 (10)	−0.0031 (8)	0.0074 (9)	−0.0057 (7)
C16	0.0689 (15)	0.0515 (12)	0.0396 (12)	0.0056 (11)	−0.0005 (11)	−0.0025 (10)
C17	0.110 (2)	0.0658 (16)	0.0388 (13)	0.0026 (15)	−0.0039 (14)	−0.0051 (12)
C18	0.135 (3)	0.0554 (15)	0.0415 (14)	−0.0112 (16)	0.0260 (17)	−0.0101 (11)
C19	0.098 (2)	0.0676 (17)	0.0699 (19)	−0.0138 (15)	0.0470 (17)	−0.0170 (14)
C20	0.0604 (15)	0.0546 (13)	0.0598 (15)	−0.0074 (11)	0.0178 (12)	−0.0099 (11)
S2	0.0619 (3)	0.0239 (2)	0.0325 (3)	0.0015 (2)	−0.0038 (2)	−0.00153 (18)
O4	0.0589 (10)	0.0428 (8)	0.0571 (9)	−0.0192 (7)	−0.0116 (7)	0.0086 (7)
O5	0.1104 (14)	0.0397 (8)	0.0414 (9)	0.0282 (8)	−0.0011 (8)	−0.0074 (6)
O6	0.0610 (9)	0.0305 (7)	0.0485 (8)	0.0107 (6)	0.0025 (7)	−0.0063 (6)
N3	0.0479 (10)	0.0271 (7)	0.0316 (8)	0.0024 (6)	0.0031 (7)	−0.0017 (6)
C21	0.0401 (11)	0.0308 (9)	0.0330 (10)	0.0023 (7)	−0.0005 (8)	0.0036 (7)
N4	0.0545 (11)	0.0441 (9)	0.0307 (9)	0.0005 (7)	0.0023 (7)	0.0013 (7)
C23	0.0561 (13)	0.0559 (13)	0.0332 (11)	0.0003 (10)	0.0026 (9)	0.0088 (9)
C24	0.0473 (12)	0.0451 (11)	0.0479 (12)	−0.0036 (9)	0.0014 (10)	0.0175 (9)
C25	0.0416 (11)	0.0301 (9)	0.0503 (12)	−0.0004 (8)	−0.0018 (9)	0.0051 (8)
C26	0.0316 (10)	0.0288 (8)	0.0373 (10)	0.0006 (7)	−0.0002 (8)	0.0001 (7)
C27	0.0369 (10)	0.0291 (9)	0.0392 (10)	−0.0010 (7)	−0.0019 (8)	−0.0042 (7)
C28	0.0424 (10)	0.0265 (8)	0.0324 (9)	0.0014 (7)	−0.0010 (8)	−0.0018 (7)
C29	0.0632 (13)	0.0293 (9)	0.0310 (10)	0.0043 (9)	−0.0030 (9)	0.0000 (8)
C30	0.0805 (17)	0.0383 (11)	0.0418 (12)	−0.0025 (10)	0.0081 (11)	0.0017 (9)
C31	0.133 (3)	0.0419 (12)	0.0476 (14)	−0.0040 (14)	0.0228 (16)	0.0059 (11)
C32	0.170 (4)	0.0582 (17)	0.0487 (16)	0.028 (2)	−0.001 (2)	0.0197 (13)
C33	0.119 (3)	0.086 (2)	0.0635 (18)	0.035 (2)	−0.0273 (18)	0.0165 (16)
C34	0.0714 (17)	0.0604 (14)	0.0548 (14)	0.0069 (12)	−0.0152 (12)	0.0078 (11)
C35	0.0615 (14)	0.0312 (9)	0.0346 (10)	0.0008 (9)	0.0086 (9)	−0.0022 (8)
C36	0.0666 (16)	0.0621 (14)	0.0587 (15)	0.0074 (12)	0.0179 (12)	0.0076 (12)
C37	0.111 (3)	0.093 (2)	0.077 (2)	0.0220 (19)	0.053 (2)	0.0210 (18)
C38	0.171 (4)	0.116 (3)	0.0408 (17)	0.009 (3)	0.033 (2)	0.0017 (17)
C39	0.144 (3)	0.137 (3)	0.0400 (16)	−0.025 (3)	0.0044 (18)	−0.0144 (18)
C40	0.0865 (19)	0.0826 (18)	0.0381 (13)	−0.0191 (15)	0.0032 (12)	−0.0114 (12)

### 2,3-Diphenyl-2,3-dihydro-4H-pyrido[3,2-e][1,3]thiazine-1,1,4-trione (2) Geometric parameters (Å, º)

**Table:**

S1—O1	1.4309 (16)	S2—O4	1.4349 (16)
S1—O2	1.4289 (16)	S2—O5	1.4268 (15)
S1—C1	1.7776 (18)	S2—C21	1.7762 (18)
S1—C8	1.8085 (19)	S2—C28	1.8037 (18)
O3—C7	1.222 (2)	O6—C27	1.218 (2)
N1—C7	1.364 (2)	N3—C27	1.364 (2)
N1—C8	1.466 (2)	N3—C28	1.473 (2)
N1—C15	1.459 (2)	N3—C35	1.447 (2)
C1—N2	1.331 (2)	C21—N4	1.327 (2)
C1—C6	1.386 (3)	C21—C26	1.386 (2)
N2—C3	1.336 (3)	N4—C23	1.329 (2)
C3—H3	0.9300	C23—H23	0.9300
C3—C4	1.373 (3)	C23—C24	1.383 (3)
C4—H4	0.9300	C24—H24	0.9300
C4—C5	1.386 (3)	C24—C25	1.379 (3)
C5—H5	0.9300	C25—H25	0.9300
C5—C6	1.395 (2)	C25—C26	1.389 (2)
C6—C7	1.506 (3)	C26—C27	1.510 (3)
C8—H8	0.9800	C28—H28	0.9800
C8—C9	1.503 (2)	C28—C29	1.510 (2)
C9—C10	1.390 (3)	C29—C30	1.379 (3)
C9—C14	1.377 (3)	C29—C34	1.390 (3)
C10—H10	0.9300	C30—H30	0.9300
C10—C11	1.376 (3)	C30—C31	1.383 (3)
C11—H11	0.9300	C31—H31	0.9300
C11—C12	1.372 (3)	C31—C32	1.365 (4)
C12—H12	0.9300	C32—H32	0.9300
C12—C13	1.372 (3)	C32—C33	1.367 (4)
C13—H13	0.9300	C33—H33	0.9300
C13—C14	1.383 (3)	C33—C34	1.382 (3)
C14—H14	0.9300	C34—H34	0.9300
C15—C16	1.367 (3)	C35—C36	1.372 (3)
C15—C20	1.379 (3)	C35—C40	1.366 (3)
C16—H16	0.9300	C36—H36	0.9300
C16—C17	1.387 (3)	C36—C37	1.397 (4)
C17—H17	0.9300	C37—H37	0.9300
C17—C18	1.356 (4)	C37—C38	1.375 (5)
C18—H18	0.9300	C38—H38	0.9300
C18—C19	1.370 (4)	C38—C39	1.353 (5)
C19—H19	0.9300	C39—H39	0.9300
C19—C20	1.397 (3)	C39—C40	1.382 (4)
C20—H20	0.9300	C40—H40	0.9300
			
O1—S1—C1	108.86 (9)	O4—S2—C21	108.44 (8)
O1—S1—C8	111.01 (9)	O4—S2—C28	111.03 (9)
O2—S1—O1	119.48 (10)	O5—S2—O4	119.14 (10)
O2—S1—C1	109.82 (9)	O5—S2—C21	109.50 (9)
O2—S1—C8	108.66 (9)	O5—S2—C28	108.74 (9)
C1—S1—C8	96.59 (8)	C21—S2—C28	97.89 (8)
C7—N1—C8	119.92 (15)	C27—N3—C28	120.15 (15)
C7—N1—C15	118.80 (14)	C27—N3—C35	118.72 (14)
C15—N1—C8	119.39 (13)	C35—N3—C28	118.90 (14)
N2—C1—S1	116.15 (14)	N4—C21—S2	115.57 (13)
N2—C1—C6	126.13 (17)	N4—C21—C26	126.60 (16)
C6—C1—S1	117.72 (14)	C26—C21—S2	117.81 (14)
C1—N2—C3	115.68 (18)	C21—N4—C23	115.95 (16)
N2—C3—H3	118.0	N4—C23—H23	118.4
N2—C3—C4	123.90 (19)	N4—C23—C24	123.15 (19)
C4—C3—H3	118.0	C24—C23—H23	118.4
C3—C4—H4	120.5	C23—C24—H24	120.4
C3—C4—C5	119.07 (18)	C25—C24—C23	119.26 (17)
C5—C4—H4	120.5	C25—C24—H24	120.4
C4—C5—H5	120.5	C24—C25—H25	120.3
C4—C5—C6	118.94 (19)	C24—C25—C26	119.32 (17)
C6—C5—H5	120.5	C26—C25—H25	120.3
C1—C6—C5	116.27 (17)	C21—C26—C25	115.71 (17)
C1—C6—C7	125.04 (15)	C21—C26—C27	124.67 (15)
C5—C6—C7	118.58 (16)	C25—C26—C27	119.46 (16)
O3—C7—N1	122.41 (17)	O6—C27—N3	122.22 (17)
O3—C7—C6	119.79 (15)	O6—C27—C26	119.29 (16)
N1—C7—C6	117.77 (15)	N3—C27—C26	118.39 (15)
S1—C8—H8	107.5	S2—C28—H28	107.6
N1—C8—S1	108.82 (12)	N3—C28—S2	107.80 (12)
N1—C8—H8	107.5	N3—C28—H28	107.6
N1—C8—C9	115.31 (14)	N3—C28—C29	115.84 (15)
C9—C8—S1	109.85 (12)	C29—C28—S2	110.23 (12)
C9—C8—H8	107.5	C29—C28—H28	107.6
C10—C9—C8	122.03 (16)	C30—C29—C28	123.24 (19)
C14—C9—C8	118.21 (16)	C30—C29—C34	119.20 (19)
C14—C9—C10	119.72 (17)	C34—C29—C28	117.46 (19)
C9—C10—H10	120.1	C29—C30—H30	119.9
C11—C10—C9	119.8 (2)	C29—C30—C31	120.2 (2)
C11—C10—H10	120.1	C31—C30—H30	119.9
C10—C11—H11	119.8	C30—C31—H31	119.8
C12—C11—C10	120.4 (2)	C32—C31—C30	120.3 (3)
C12—C11—H11	119.8	C32—C31—H31	119.8
C11—C12—H12	120.0	C31—C32—H32	119.9
C11—C12—C13	120.03 (19)	C31—C32—C33	120.1 (3)
C13—C12—H12	120.0	C33—C32—H32	119.9
C12—C13—H13	119.9	C32—C33—H33	119.8
C12—C13—C14	120.3 (2)	C32—C33—C34	120.4 (3)
C14—C13—H13	119.9	C34—C33—H33	119.8
C9—C14—C13	119.8 (2)	C29—C34—H34	120.1
C9—C14—H14	120.1	C33—C34—C29	119.8 (3)
C13—C14—H14	120.1	C33—C34—H34	120.1
C16—C15—N1	119.61 (18)	C36—C35—N3	119.09 (19)
C16—C15—C20	120.9 (2)	C40—C35—N3	119.53 (19)
C20—C15—N1	119.38 (18)	C40—C35—C36	121.4 (2)
C15—C16—H16	120.4	C35—C36—H36	120.6
C15—C16—C17	119.2 (2)	C35—C36—C37	118.7 (3)
C17—C16—H16	120.4	C37—C36—H36	120.6
C16—C17—H17	119.7	C36—C37—H37	120.2
C18—C17—C16	120.6 (3)	C38—C37—C36	119.6 (3)
C18—C17—H17	119.7	C38—C37—H37	120.2
C17—C18—H18	119.7	C37—C38—H38	119.8
C17—C18—C19	120.5 (2)	C39—C38—C37	120.4 (3)
C19—C18—H18	119.7	C39—C38—H38	119.8
C18—C19—H19	120.1	C38—C39—H39	119.6
C18—C19—C20	119.8 (3)	C38—C39—C40	120.8 (3)
C20—C19—H19	120.1	C40—C39—H39	119.6
C15—C20—C19	118.9 (2)	C35—C40—C39	119.0 (3)
C15—C20—H20	120.5	C35—C40—H40	120.5
C19—C20—H20	120.5	C39—C40—H40	120.5
			
S1—C1—N2—C3	179.14 (15)	S2—C21—N4—C23	177.70 (14)
S1—C1—C6—C5	−179.38 (14)	S2—C21—C26—C25	−178.19 (14)
S1—C1—C6—C7	4.6 (3)	S2—C21—C26—C27	−2.7 (2)
S1—C8—C9—C10	64.1 (2)	S2—C28—C29—C30	−56.3 (2)
S1—C8—C9—C14	−113.40 (17)	S2—C28—C29—C34	119.99 (18)
O1—S1—C1—N2	97.46 (17)	O4—S2—C21—N4	−95.33 (16)
O1—S1—C1—C6	−82.47 (16)	O4—S2—C21—C26	82.93 (16)
O1—S1—C8—N1	52.60 (14)	O4—S2—C28—N3	−53.75 (14)
O1—S1—C8—C9	−74.51 (14)	O4—S2—C28—C29	73.56 (15)
O2—S1—C1—N2	−35.07 (19)	O5—S2—C21—N4	36.17 (18)
O2—S1—C1—C6	145.00 (15)	O5—S2—C21—C26	−145.57 (15)
O2—S1—C8—N1	−174.07 (12)	O5—S2—C28—N3	173.30 (12)
O2—S1—C8—C9	58.82 (15)	O5—S2—C28—C29	−59.39 (16)
N1—C8—C9—C10	−59.3 (2)	N3—C28—C29—C30	66.4 (2)
N1—C8—C9—C14	123.22 (18)	N3—C28—C29—C34	−117.3 (2)
N1—C15—C16—C17	174.64 (19)	N3—C35—C36—C37	176.9 (2)
N1—C15—C20—C19	−175.11 (19)	N3—C35—C40—C39	−177.0 (2)
C1—S1—C8—N1	−60.54 (13)	C21—S2—C28—N3	59.54 (13)
C1—S1—C8—C9	172.35 (13)	C21—S2—C28—C29	−173.14 (14)
C1—N2—C3—C4	0.1 (3)	C21—N4—C23—C24	0.9 (3)
C1—C6—C7—O3	157.04 (18)	C21—C26—C27—O6	−159.73 (18)
C1—C6—C7—N1	−20.8 (3)	C21—C26—C27—N3	16.7 (3)
N2—C1—C6—C5	0.7 (3)	N4—C21—C26—C25	−0.1 (3)
N2—C1—C6—C7	−175.37 (18)	N4—C21—C26—C27	175.31 (18)
N2—C3—C4—C5	0.9 (3)	N4—C23—C24—C25	−0.9 (3)
C3—C4—C5—C6	−1.1 (3)	C23—C24—C25—C26	0.4 (3)
C4—C5—C6—C1	0.4 (3)	C24—C25—C26—C21	0.1 (3)
C4—C5—C6—C7	176.70 (17)	C24—C25—C26—C27	−175.55 (17)
C5—C6—C7—O3	−18.9 (3)	C25—C26—C27—O6	15.6 (3)
C5—C6—C7—N1	163.18 (16)	C25—C26—C27—N3	−167.98 (16)
C6—C1—N2—C3	−0.9 (3)	C26—C21—N4—C23	−0.4 (3)
C7—N1—C8—S1	57.44 (18)	C27—N3—C28—S2	−58.41 (19)
C7—N1—C8—C9	−178.63 (15)	C27—N3—C28—C29	177.60 (16)
C7—N1—C15—C16	99.0 (2)	C27—N3—C35—C36	89.0 (2)
C7—N1—C15—C20	−84.2 (2)	C27—N3—C35—C40	−92.1 (2)
C8—S1—C1—N2	−147.65 (16)	C28—S2—C21—N4	149.32 (15)
C8—S1—C1—C6	32.42 (16)	C28—S2—C21—C26	−32.42 (16)
C8—N1—C7—O3	167.93 (17)	C28—N3—C27—O6	−165.63 (17)
C8—N1—C7—C6	−14.3 (2)	C28—N3—C27—C26	18.0 (2)
C8—N1—C15—C16	−65.4 (2)	C28—N3—C35—C36	−107.9 (2)
C8—N1—C15—C20	111.5 (2)	C28—N3—C35—C40	70.9 (2)
C8—C9—C10—C11	−178.63 (18)	C28—C29—C30—C31	175.96 (19)
C8—C9—C14—C13	178.46 (19)	C28—C29—C34—C33	−176.8 (2)
C9—C10—C11—C12	0.7 (3)	C29—C30—C31—C32	0.6 (4)
C10—C9—C14—C13	0.9 (3)	C30—C29—C34—C33	−0.4 (3)
C10—C11—C12—C13	0.0 (3)	C30—C31—C32—C33	−0.2 (4)
C11—C12—C13—C14	−0.3 (4)	C31—C32—C33—C34	−0.4 (5)
C12—C13—C14—C9	−0.2 (3)	C32—C33—C34—C29	0.7 (4)
C14—C9—C10—C11	−1.2 (3)	C34—C29—C30—C31	−0.3 (3)
C15—N1—C7—O3	3.7 (3)	C35—N3—C27—O6	−2.8 (3)
C15—N1—C7—C6	−178.52 (15)	C35—N3—C27—C26	−179.13 (16)
C15—N1—C8—S1	−138.38 (14)	C35—N3—C28—S2	138.78 (14)
C15—N1—C8—C9	−14.5 (2)	C35—N3—C28—C29	14.8 (2)
C15—C16—C17—C18	1.1 (4)	C35—C36—C37—C38	0.3 (4)
C16—C15—C20—C19	1.7 (3)	C36—C35—C40—C39	1.9 (4)
C16—C17—C18—C19	0.4 (4)	C36—C37—C38—C39	1.3 (5)
C17—C18—C19—C20	−0.9 (4)	C37—C38—C39—C40	−1.4 (6)
C18—C19—C20—C15	−0.2 (3)	C38—C39—C40—C35	−0.2 (5)
C20—C15—C16—C17	−2.2 (3)	C40—C35—C36—C37	−1.9 (3)

### 2,3-Diphenyl-2,3-dihydro-4H-pyrido[3,2-e][1,3]thiazine-1,1,4-trione (2) Hydrogen-bond geometry (Å, º)

**Table:**

*D*—H···*A*	*D*—H	H···*A*	*D*···*A*	*D*—H···*A*
C3—H3···O5^i^	0.93	2.56	3.234 (3)	130
C5—H5···O4^ii^	0.93	2.69	3.388 (2)	132
C8—H8···O6^ii^	0.98	2.31	3.089 (2)	136
C16—H16···O6^ii^	0.93	2.71	3.466 (3)	139
C20—H20···O6	0.93	2.70	3.496 (3)	145
C25—H25···O1	0.93	2.75	3.463 (3)	135
C28—H28···O3	0.98	2.37	3.126 (2)	134
C36—H36···O3^iii^	0.93	2.64	3.381 (3)	137
C40—H40···O3	0.93	2.68	3.439 (3)	140

Symmetry codes: (i) −*x*+1, −*y*+1, −*z*+1; (ii) *x*+1, *y*, *z*; (iii) *x*−1, *y*, *z*.
